# The effect of the COVID-19 pandemic on paediatric cancer care: lessons learnt from a major paediatric oncology department in Turkey

**DOI:** 10.3332/ecancer.2021.1172

**Published:** 2021-01-14

**Authors:** Mustafa Tezer Kutluk, Fahad Ahmed, Meral Kirazlı, İnci Yaman Bajin, Eren Müngen, Saniye Ekinci, Ferah Yıldız

**Affiliations:** 1 Department of Pediatric Oncology, Hacettepe University Faculty of Medicine & Cancer Institute, 06100 Ankara, Turkey; 2 Department of Pediatric Surgery, Hacettepe University Faculty of Medicine, 06100 Ankara, Turkey; 3 Department of Radiation Oncology, Hacettepe University Faculty of Medicine, 06100 Ankara, Turkey

**Keywords:** COVID-19 pandemic, pediatric cancer, delayed diagnosis, chemotherapy, radiotherapy, surgical procedures

## Abstract

The COVID-19 pandemic has disrupted cancer care. An audit at a major Paediatric Oncology Department in Turkey was performed to determine its impact on paediatric cancer care. A comparison was made among the number of daily paediatric cancer patients, diagnostic and treatment procedures. The data for the ‘COVID-19 period’ (10 March to 31 October 2020) were compared with the corresponding ‘prior year control period’ (10 March to 31 October 2019). Moreover, presentation delay (duration between first symptoms to healthcare visit) was calculated for new cases. The findings indicate that the mean 34.7 outpatients per day during ‘COVID-19 period’ was significantly lower than the ‘prior year control period’ (52.2). There were 17.7 inpatients per day during the ‘COVID-19 period’ which was significantly lower than 23.8 inpatients per day during the ‘prior year control period’. Significant reduction in the daily mean number of patients undergoing chemotherapy, radiotherapy, surgery and imaging studies during the ‘COVID-19 period’ was also evident. A negative trend in the diagnosis of new paediatric cancers was evident with 128 new cancer cases during the ‘COVID-19 period’, whereas the corresponding number was 212 for the ‘prior year control period’. The presentation delay (median 31 days) remain unchanged during the ‘COVID-19 period’. The findings suggest significant damage to paediatric cancer care during the COVID-19 pandemic. Appropriate obligatory actions by oncology societies and policymakers can minimise longer term negative impacts.

## Introduction

After the occurrence of the first cases of COVID-19 in China in December [1], Turkey faced the first COVID-19 case on 10 March [2], followed by a major pandemic and crisis within a few weeks around the globe. Every health care organisation in the nation started to re-organise their services to provide the best available care to the people based on the information and rules from the local, regional and international sources. The information and experience of the global medical community started to travel around the world with rocket speed to control the dissemination of infection and to provide guidance on COVID-19 infection prevention and control. Although elective appointments, procedures and other investigations were advised to postpone, not much was spoken about the care of non-coronavirus diseases in the beginning since the fear about the capacity of health care and the burden of infected cases was unpredicted. On 30 January 2020, the World Health Organization (WHO) announced COVID-19 as a public health emergency of international concern [3], followed by the characterisation of COVID-19 as a pandemic [4]. However, the Turkish people’s concern about the pandemic was not high until the first case was reported in the country. The Ministry of Health (MoH) in Turkey established the Scientific Board to advise the Government to address the pandemic. The Turkish health ministry started to take actions in the early days of the outbreak. Strict measures have been adopted nationwide to protect people’s safety. This includes closure of borders and domestic travel restrictions, use of personal protective equipment and maintenance of social distancing, closure of schools, religious places, restaurants and shopping malls, cancellation of the social gatherings and meetings, partial stay home orders and weekend lock-downs in major cities during the following weeks; healthcare providers also witnessed huge number of regulations, mandatory precautions and recommendations at the national level by the MoH, the University level by the President of the University, Hospital Management and Infection Control Committee [5–7].

Early reports suggested that cancer was a risk factor for the severity of infection with a hazard ratio of 3.56 among adults with cancer [8], and a leukaemia case with COVID-19 infection was reported [9]. A meta-analysis of 32 studies including 46,499 patients (1,776 with cancer) showed that risk ratio for all-cause mortality among cancer patients was 1.66 and 1.56 for intensive care unit admission [10]. The leaders of the paediatric oncology community came with early proposals on how to manage the situation for paediatric cancer patients [11, 12]. The *American Society of Clinical Oncology*, like many other major organisations, started to share the expert views and evidences with the rest of the world [13]. It became a real challenge for us as the nation’s biggest paediatric oncology center with a focus to continue to treat our patients and not to cause any harm due to delay in cancer treatment in addition to protecting them from the new Coronavirus infection. We quickly implemented the policies from the hospital management, MoH and international scientific communities. However, the management of such an extraordinary situation had many difficulties without having strong evidences in the early days of the pandemic. This study aimed to analyse how this pandemic affected our routine paediatric oncology practices, in relation to the national and international reactions to control COVID-19 infection and share the lessons learnt from this experience.

## Materials and Methods

### Study design and data sources

In this retrospective study, we extracted clinical data for consecutive paediatric cancer patients (age ≤ 18) admitted to the paediatric oncology department from 01 January to 31 October, for the years of 2019 and 2020, from patients’ medical records and hospital information system. The data were checked for the accuracy and the identity of the patient was kept confidential in the study dataset. The daily number of COVID-19 cases and mortalities due to COVID-19 during the study period were also obtained from the official Turkish MoH COVID-19 website [2]. This study was approved by the Turkish Ministry of Health Scientific Research Projects Review Committee and the Institutional Review Board of Hacettepe University.

### Study periods

To perform comparative analyses, the study period was grouped into two different time spans; ‘*COVID-19 period’,* from 10 March 2020 (the first confirmed case of COVID-19 in Turkey) to 31 October 2020 (the end date for this analysis), and the ‘*prior year control period’,* from 10 March 2019 to 31 October 2019 (a corresponding period to COVID-19 during the previous year). There are 236 calendar days in each of these periods whereas 159 working days (excluding weekend and public holiday) in the COVID-19 period and 156 working days in the prior year control period.

### Variables

The following variables were retrieved retrospectively from the database. Outpatient visits (daily number of paediatric cancer patients visiting the outpatient department.), in-patients (daily number of paediatric cancer patients at the inpatient department for treatment that requires at least one overnight stay), surgical procedures (daily number of paediatric cancer patients undergoing any procedure including biopsy, tumour excisions, insert subcutaneous port and other surgical procedures), chemotherapies (daily number of paediatric cancer patients having intravenous treatment with anticancer drugs at the oncology department both outpatient and inpatient), imaging studies (daily number of paediatric cancer patients undergoing X-ray, ultrasound imaging, CT-scan, MRI, PET-CT for screening and detection of tumour, tumour related illness and complications) and radiotherapy (daily number of paediatric cancer patients undergoing treatment with radiation).

The number of ‘*new cases’* was defined as referred to the department for diagnosis and treatment. For the new cases, information regarding the nature of presenting symptoms attributable to cancer, the date of first concern from patients/parents/caregivers and the date of first presentation at Hacettepe University Hospital due to that symptom were also retrieved. This information was used to calculate the ‘*presentation delay*’ which is defined as the number of days between the first symptom and first presentation to the Department of Paediatric Oncology at Hacettepe University. The diagnosis of the new cases was classified according to the International Classification for Childhood Cancer [14].

### Statistical analysis

The distribution of the data was verified for normality. The values are reported as mean and standard deviation or median and interquartile range. To evaluate the difference in variables during study period and control period, independent *t-*test and Mann–Whitney’s U test were performed as appropriate. Values were considered significantly different at *p* < 0.05. Statistical analysis was performed using SPSS 23 (IBM Corporation, NY, USA) and Microsoft Excel.

## Results

There were a total of 5,515 visits to the paediatric oncology outpatient department during the ‘COVID-19 period’. This accounts for a mean of 34.7 patients per day and was significantly lower compared to ‘the prior year control period’ (50.2 patients per day; *p* < 0.00001). Accordingly, a 29.6% decrease in total number of outpatients was noted during the COVID-19 period.

The mean number of inpatients during the ‘COVID-19 period’ was 17.7 per day. This was also significantly lower than ‘the prior year control period’ (23.8 patients per day; *p* < 0.00001). The decrease in total number of inpatients during the COVID-19 period was 25.5%.

The significant reduction in the daily mean number of patients undergoing chemotherapy (14.2 patients per day), radiotherapy (6.0 patients per day), surgery (1.6 patients per day) and imaging studies (12.5 patients per day) during the ‘COVID-19 period’ was also evident in Table 1.

The percentage decreases were; 20.8% for chemotherapy applications, 9.3% for radiotherapy applications, 30.9% for surgical procedures, 36.5% for imaging studies, compared to the prior year control period.

**Figure 1.**
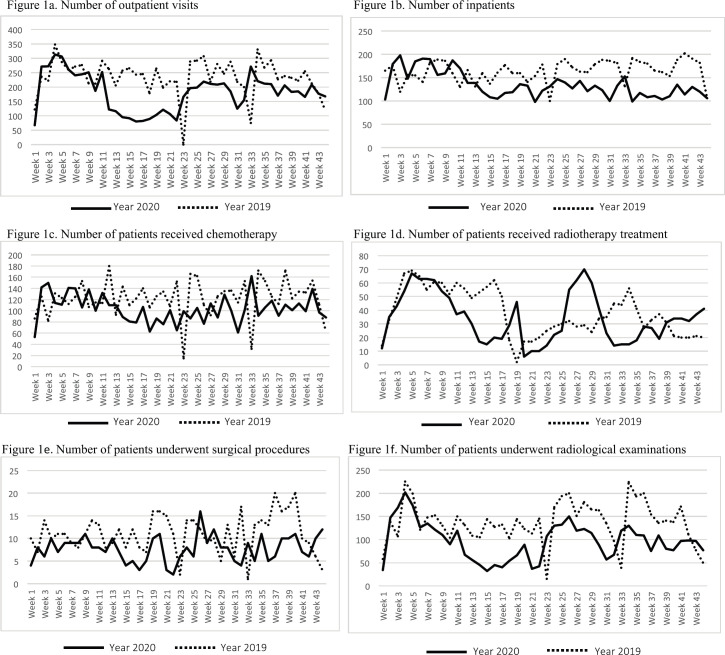
The distribution of patients by calendar week during the years 2019 and 2020. (a): Number of outpatient visits; (b): number of inpatients; (c): number of patients received chemotherapy; (d): number of patients received radiotherapy treatment; (e): number of patients underwent surgical procedures; (f): number of patients underwent radiological examinations. (a–f): The number of patients per calendar week is based on the total number of patients in the 1 to 44 calendar weeks of the year 2019 and 2020. Weeks 11, 17 and 23 of the year 2020 are corresponding to the start of the COVID-19 outbreak, partial lock-down and normalisation in Turkey, respectively. Weeks 23 and 33 of the year 2019 likewise weeks 22 and 31 of the year 2020 are corresponding to religious holidays in Turkey.

The distribution of paediatric cancer patients by calender week during the years 2019 and 2020 was examined in Figure 1. It is clear that since the outset of the pandemic on 10 March 2020 (11th week), the number of patients has decreased. Although some increase is evident after the normalisation on 1 June 2020 (23rd week), COVID-19 has resulted in an almost unprecedented drop in all areas of cancer care at our centre.

**Figure 2.**
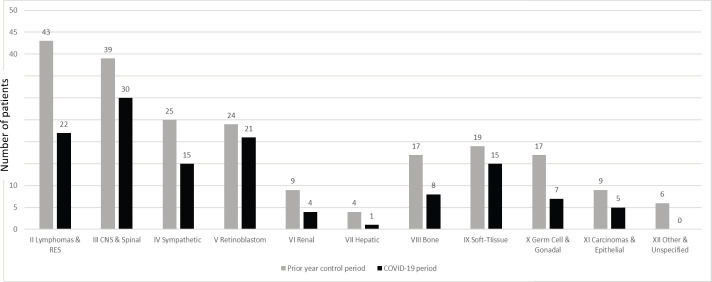
Number of new cancer diagnoses during ‘COVID-19 period’ (10 March to 31 October 2020) and ‘prior year control period’ (10 March to 31 October 2019). The names and numbers of cancer are listed according to the International Classification of Childhood Cancer third edition. (II Lymphomas and Reticuloendothelial Neoplasm, III CNS and Miscellaneous Intracranial & Intraspinal Neoplasm, IV Sympathetic Nervous System Tumors, V Retinoblastom, VI Renal Tumors, VII Hepatic Tumors, VIII Malignant Bone Tumors, IX Soft-tissue Sarcomas, X Germ Cell, Trophoblastic & Other Gonadal Neoplasm, XI Carcinomas, and Other Malignant Epithelial Neoplasm, XII Other and Unspecified Malignant Neoplasm). There were a total of 128 new cases of paediatric cancer diagnosed at Hacettepe university oncology hospital in the period between 10 March to 31 October 2019, the corresponding number for the period between 10 March 2020 and 31 October 2019, was 212.

**Figure 3.**
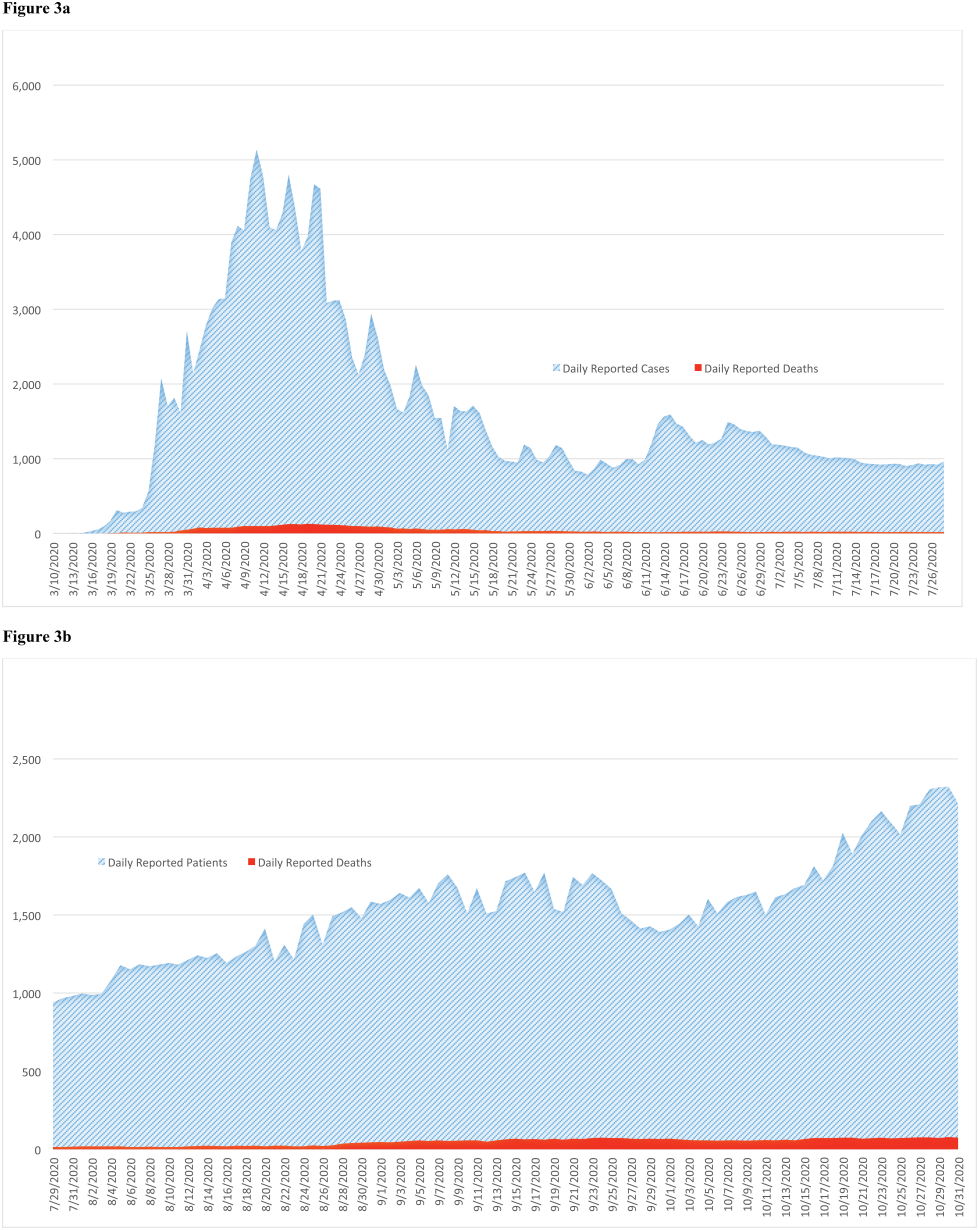
The COVID-19 pandemic in Turkey. (a): The daily number of COVID-19 cases and deaths. (b): The daily number of COVID-19 patients and deaths. SARS-CoV-2 virus (COVID-19) officially reached Turkey on 10 March 2020, when the first case was detected in Istanbul. The first death due to COVID-19 occurred in Turkey on 17 March 2020. By 1 April 2020, it was confirmed that COVID-19 had spread all over the country. The Turkish Ministry of Health reported all new cases regardless of symptoms till 28 July 2020. Between 29 July 2020 and 24 November 2020, only new symptomatic patients (exclude asymptomatic cases) were included in the announcement released to the public. Source: Ministry of Health’s COVID-19 website [2].

The number of new cases with paediatric cancers reduced considerably during the COVID-19 period (128 cases) compared to the prior year control period (212 cases). Sharp declines occurred among all cancer types, these ranged from 21.5% for soft-tissue sarcomas to 100% for other and unspecified malignant neoplasm (Figure 2). The comparison of presentation delay of new cases is presented in Table 2. It is interesting to note that the median number of days for presentation delay during the COVID-19 period and control periods was identical (or unchanged) at 31 days. Figure 3 presents daily number of cases and mortalities due to COVID-19 during the study period. The MoH changed the reporting of the data shared daily on 29 July [15]. The daily number of new ‘cases’ (all testing positive) was reported until 29 July (Figure 3a), then they started to report the daily number of new ‘patients’ (only symptomatic cases) (Figure 3b). The peak of the cases was on 11 April 2020 with 5,138 positive cases, whereas the peak of ‘patients’ was observed on 30 October 2020 with 2,322 symptomatic new cases. As of 31 October 2020, 10,252 lives have been lost to COVID-19 in Turkey. The highest single-day mortality was observed on 19 April 2020 (127 deaths).

As per MoH and hospital guidelines, COVID-19 tests were performed at our department for the suspected cases with COVID-19 symptoms or history of contact with a known case or international travel. After 4 September 2020, a COVID-19 polymerase chain reaction (PCR) test became a requirement for paediatric cancer patients to be admitted to the ward. By the time of this writing, nine children were diagnosed with COVID-19 either inpatient or outpatient. One died from pneumonia, the course was mild in the other patients and no major delays in scheduled chemotherapy occurred. Ten healthcare staff at the department caught the infection, luckily with no serious morbidity or mortality.

Table 3 shows the the policies and actions taken by the government. Table 4 summarises the actions taken by the Department of Paediatric Oncology during the COVID-19 pandemic. In addition to the rules and regulations at the national level and hospital level, a management mechanism was established as shown in Table 4. The major adaptations to the crisis were the postponement of the elective appointments and continuation of the on-going treatments, careful monitoring of the situation, and strict measurements on the prevention and control of infection to protect cancer patients.

## Discussion

While the first Coronavirus case was reported in China in early December [1], the rest of the World did not worry that much about the COVID-19 issue and did not expect what was going to happen during the following months. During the 2nd week of January, a total of 366 hospitalised children were tested and only six cases had COVID-19 in a Wuhan Hospital [16]. A nationwide data of 2,143 paediatric suspected patients and confirmed COVID-19 positive cases were reported by the Chinese Center for Disease Control and Prevention between 16 January and 8 February 2020 [17]. A retrospective analysis in two major hospitals in Ankara, Turkey reported 220 (8.6%) confirmed COVID-19 cases among 2,530 children applied with the suspicion of COVID-19 infection. One hundred and ninety-nine (90.5%) had no underlying disease and only two cases had underlying malignant disease [18]. A report from US showed 1,039,464 out of 9,037,991 (11.5%) total COVID-19 cases were children until 12 November 2020 since the beginning of the outbreak. Only 133 mortalities were reported from 42 states and New York City which makes 0.01% of children with COVID-19 infection [19]. An early report from China by Liang W *et al* [8] presented that 1% of 1,590 COVID-19 cases had a history of cancer, higher than the expected numbers for the overall Chinese population (0.29%). The first reported case was a child with leukaemia from China in March 2020 [9]. Then multiple cases of paediatric cancer with COVID-19 infection were reported in the literature [20–22]. Before the global oncology community started to publish sporadic reports in March and April 2020, the WHO made a declaration of emergency on 30 January [3]. However, most of the health care professionals and the public were not much concerned at the time of the WHO declaration; since during the previous years, Severe Acute Respiratory Syndrome (SARS), Middle East Respiratory Syndrome (MERS), Ebola, Dengi and other regional outbreaks made them accustomed to hear such news. With the passage of time, COVID-19 turned into a global crisis including health care,—economy, political priorities and actions. The information started to accumulate relatively slowly for paediatric cancers compared to adult cancers. Boulad *et al* [21] reported 2.5% test positivity among asymptomatic paediatric cancer patients and low morbidity (5%) of COVID-19 infection in paediatric cancer patients. In other reports, it was shown that COVID-19 was showing a mild course in children [20, 23]. However, it was still a challenge to manage the situation due to the uncertainity of this extraordinary situation, lack of knowledge, experience and unpreparedness for the rapid progression of the pandemic, along with the weakness of healthcare systems both in developing and developed countries [11, 12, 24, 25].

In addition to the strict measurements at the national level, on the 17 of March 2020, we started to postpone elective routine appointments at the Paediatric Oncology Department. The entire staff attended morning departmental meetings and were briefed about the rules and regulations and implementation of these at department level. From March to October 2020, we carefully watched the strategies and recommendations from around the world focusing on how to manage the situation. Most of the strategies at regional and international level mainly focused on preventing cancer patients from catching the infection, handling of the suspected cases, management of the cases with COVID-19, alignment with the hospital and national rules, managing the human resources, burden on the healthcare system, the financial barriers, communication among the governing bodies, public communication and psychosocial issues [11, 12, 20, 24, 26–31].

The International Society of Paediatric Oncology (SIOP) gave out early advice [11] and structured recommendations to adapt cancer services including diagnosis and treatment [12]. Moreover, the importance of the continuation of paediatric cancer care was emphasised. ASCO also published recommendations and guidelines regarding cancer care during the pandemic [13, 32]. The oncology community is aware of the ambitious goal of the WHO Global Initiative for Childhood Cancer to increase the survival up to 60% in low-middle income countries by 2030 [33]. Nevertheless, it is difficult to predict what the long-term effects of the pandemic will be on cancer care.

When we looked closer to the numbers at our department, the drop in major departmental functions was important to us to monitor the changes for the proper use of our human capacity and resources. COVID-19 services were not located in the oncology building, but in another building of the large hospitals complex at our university; strict measurements of the hospital mangament protected the cancer patients, care givers and staff from contamination. We do not think the decreases in the treatments weres due to treatment modifications since we only postponed the appointments for off-therapy patients, and never cancelled any ongoing treatments. However, due to uncertainity and different sources of information, the numbers of admissions, surgery, chemotherapy, radiotherapy decreased significantly as we reported in this study. Starting from mid-March, major actions were taken by the government and by the department (See Tables 3 and 4). Based on these regulations, it seems that Turkey has a low mortality rate comparing with some other countries. As of 31 October 2020, the total number of COVID-19 patients was 375,367 and 10,252 deaths were reported. The case fatality rate was 2.7% in Turkey [2, 34].

During May and June 2020, the global oncology community started to discuss the delays in diagnosis due to fears, lack of access to care and other reasons. An 8-week analysis from six paediatric haematology and oncology centers in Lombardia, Italy, displayed 21 cases within 286 patients tested for COVID-19 and the authors suggested that chemotherapy may continue without major adjustments [35]. Carai *et al* [36] reported the indirect effects of the pandemic that could cause delays and pointed out the importance of the continuation of cancer treatment. It was reported that the Children’s Hospital of Philadelphia did not see any patients with new leukaemia for 35 days in March–April 2020 which was very unusual [37]. Gampel *et al* [20] also discussed the prospective that COVID-19 may not pose a greater risk compared to other viral infections. So, we should not be in a discussion of COVID-19 versus Cancer anyway**,** we must have an ongoing regular analysis of benefits and risks of cancer patients. To evaluate this, we carefully examined the new cancer patient admissions and found a significant drop in new admissions. We believe the messages from the health authories to the public should be adjusted for all non-Coronavirus life threatening diseases including cancer. However, the time between the first symptom and the first presentation to the Department of Paediatric Oncology was exactly the same for the COVID-19 period and the prior control period. This could be due to our hospital’s reputation for the treatment of paediatric cancers, and having a separate oncology hospital building within the university hospital complexes. Since there is a major decrease in new diagnoses during the study period, we assume that there is a possibility of the delayed diagnosis in coming years or in larger nationwide analysis. More in-depth analysis is needed to identify the impact of the pandemic on the delay of paediatric cancer diagnosis. The reason for the low number of COVID-19 cases in the largest Paediatric Oncology Department in Turkey was mainly because of having an efficient triage in practice, and proper training of staff and patients/care givers. The health literacy level of care givers, being trained at the time of cancer diagnosis, made their adaptation to the rules easier, with a higher compliance rate. We believe these were the principal factors for protection of our cancer patients from COVID-19 infection. It is important to know that all nine cases had been diagnosed after the normalisation in 1 June 2020. So, strict measures are needed to protect cancer patients during the following waves of the pandemic. As of late November 2020, the goverment reinstated the major restrictions to control the second wave in Turkey (Table 3).

Maringe *et al* [38] evaluated the impact of diagnostic delay on the survival of four major cancers; breast, colorectal, lung and oseophageal and projected 8%–10%, 15%–17%, 5% and 6% increase in mortality, respectively, for these cancers. Timely diagnosis and treatment are important; cancer care should not be neglected during the pandemic. The governmental bodies, international organisations and other stakeholders must be aware of the risks with regard to non-communicable diseases including cancers and should make plans to avoid post-COVID-19 disasters. Omarini *et al* [39] also discussed the issue by saying not to postpone but to decide wisely after analysing the data of 11,257 adult cancer patients with 22% total mortality, it was found that only nine cases (0.71%) were infected with COVID-19. Sud *et al* [40] from the UK estimated the delay of 3–6 months in cancer surgery would mitigate 19%–43% of life years gained, respectively, by hospitalisation of equal volume admission of COVID-19 cases [40]. Cancer Core Europe has published their recommendation on systemic chemotherapy, surgery and radiotherapy with priority levels to secure the continuity of the care [41]. The reports on cancer care during the COVID-19 pandemic period strongly pointed out the benefits and risks of delaying treatment and weaknesses in care and discussed the proper prioritisation for cancer care with evidence based policies and decisions [42–47]. St. Jude Children’s Research Hospital and SIOP have started a global COVID-19 registry, as of 03 December 2020, a total of 1,122 COVID-19 cases were reported from 43 countries. The data also showed that chemotherapy was reduced in 6.2%, and witheld for 37%. Radiotherapy was delayed for 1.6%, surgery was delayed for 2.2% and 4.3% died of COVID-19 infection [48]. COVID-19 has also affected cancer research. A study from Europe showed that COVID-19 significantly affected early-phase clinical trials. The patient recruitment dropped 61% compared with the recruitment period of 2019 [49].

The public should be informed not to delay seeking care for potentially life-threatening illnesses. The Turkish Radiation Oncology group reported a 50% decrease in new patient admission during the first 2 months of the pandemic in 109 departments [50]. The adult oncology department of our hospital also reported a significant decrease in the number of outpatients during the COVID-19 pandemic [51].

Since COVID-19 cases were taken care of in isolated special units of the hospital complex, our oncology hospital was functioning like a sanctuary for cancer patients both in the adult and paediatric populations. A study on cancer patients demonstrated a high rate of nosocomial transmission of COVID-19 infection, with increased mortality. It was emphasised that cancer patients must be managed in COVID-free units [52]. Between mid-March and mid-April 2020 as the COVID-19 cases were increasing steadily, the national health authorities were announcing the cancellation of elective appointments. This gave a perception in some non-COVID-19 patients with serious health conditions that they should not visit hospital. During this extraordinary situation, we wanted to monitor the impact of the pandemic on our routine work, along with the possible damage and potential threats. We tried to point out the regulations for the management of present and future situations. We considered this to be important since the duration of the pandemic is unknown. We tried to improve the management capacity within the department for this crisis and being prepared for any future potential crisis. We wanted to share our experience with the rest of the world, by managing the issue at the major paediatric oncology department in Turkey. In the end, we believe that we have managed the situation properly to continue the treatment of current on-therapy cases till now. We have started online tumour boards, grand rounds and education. However, it was obvious that training and research was also significantly affected. We used the advantage of having more time for intra-departmental training, maintaining a faculty club for scientific discussions and research planning. Although the mortality in children is much less than in the adult population, it is still higher in children with underlying disease compared to those without underlying disease. During the normalisation, children with underlying disease must be handled carefully and so it is a great challenge for us to be ready for the potential second and/or following waves and focus on the post-Coronavirus period, to prevent and fix the damages due to this pandemic. We have to monitor carefully how the survival rates as the final endpoint will be affected by this global crisis.

A recent systematic review has shown the paucity of literature for COVID-19 in paediatric cancer patients [53]. We hope our study will be helpful for paediatric cancer service providers. We will continue to monitor the impact of COVID-19 at our department. Let us not forget it is not only COVID-19 or cancer, there may be potential global damages to child health as risks reported by international organisations. The current COVID-19 crisis caused the pause or postponement of vaccination in several countries. It was estimated that 117 million children in 37 countries missed the measles vaccination which affects global child health [54]. The development of the COVID-19 vaccine is a top priority issue for global control of the pandemic. However, the focus on children is neglected. In 17 November, the American Academy of Pediatrics made a call to manufacturers to include children in their COVID-19 vaccine trials. This call was supported by some other medical associations as well [55]. When children are included in vaccine trials, the next step will be ‘how to protect paediatric cancer patients’ due to their risk from underlying disease and immunosuppressive treatment**.**

## Conclusion

Although strict measures have been taken by many nations, as of 3 December 2020, the world has 63,965,092 confirmed cases of COVID-19 and 1,488,120 deaths [56] and the pandemic still continues. Oncology professionals should not only worry for today but also make plans for the post-Coronavirus period. It affects not only cancer care but also research and education which are critically important for planning the future.

## Abbreviations key

MoH: Ministry of Health
SIOP: International Society of Paediatric Oncology
WHO: World Health Organization

## Conflict of interest

The authors declare that there is no conflict of interest regarding the publication of this article.

## Author contributions

All authors have contributed equally.

## Funding statement

M. Tezer Kutluk, Fahad Ahmed and Meral Kirazlı receive funding from the UK Research and Innovation Global Challenges Research Fund (GCRF) grant Research for Health in Conflict (R4HC-MENA) (ES/P010962/1).

## Figures and Tables

**Table 1.** Comparison of study variables for paediatric cancer patients at Hacettepe University during two different periods.

Characteristics	COVID-19 period 10 March to 31 October 2020	Prior year control period 10 March to 31 October 2019
	Total No. of patients Mean^a^ (SD)	Total No. of patients Mean^a^ (SD)
Outpatient visits^b^	5,515 34.68 (15.91)	7,838 50.24 (17.31)
*t*-test		*t*(313) = 8.30582, ***p* < 0.00001**
Inpatients^c^	4,184 17.73 (3.49)	5,617 23.80 (4.76)
*t*-test		*t*(470) = 15.78908 ***p* < 0.00001**
Chemotherapies^c^	3,349 14.19 (7.11)	4,229 17.92 (9.54)
*t*-test		*t*(470) = 4.811, ***p* < 0.00001**
Radiotherapy^b^	960 6.04 (3.22)	1,059 6.79 (3.05)
*t*-test		*t*(313)= 2.12235, ***p* = 0.034594**
Surgical procedures^b^	254 1.59 (1.23)	368 2.36 (1.79)
*t*-test		*t*(313) = 4.39426, ***p* = 0.000015**
Imaging studies^c^	2,940 12.46 (9.52)	4,630 19.62 (14.47)
*t*-test		*t*(470) = 6.35059, ***p* < 0.00001**

SD; Standard deviation

a Mean number of patients per day

b 159 working days (excluding weekend and public holiday) in COVID-19 period and 156 working days in the prior year control period.

c 236 calendar days (including weekend and public holidays) in both periods

Values set in **bold** indicate *p*-values < 0.05

**Table 2.** Number of new cases with paediatric cancer and comparison of presentation delay of new cases in the Department of Paediatric Oncology at Hacettepe University during two different periods.

Characteristics	COVID-19 period 10 March to 31 October 2020	Prior year control period 10 March to 31 October 2019
Number of new cases	128	212
Presentation delay Minimum Days–Maximum Days Median (IQR) Days *U*-value,* Z*-score *p*-values	1–784 31 (73.5)	1–730 31 (76) 12,389, −1.34205 0.18024

IQR; Interquartile range

**Table 3.** The major decisions and precautions in Turkey.

December 2020	First corona virus cases in China
30 January 2020	WHO announcement of emergency situation
03 February 2020	Travel ban to/from China
23 February 2020	Borders with Iran closed and travel ban
29 February 2020	Travel ban to/from Italia, South Korea, Iraq
10 March 2020	First corona case in Turkey
11 March 2020	WHO announcement of pandemia
12 March 2020	Ban on international travels for government officers
	All sports game cancelled
13 March 2020	US announced of emergency situation
16 March 2020	All schools are closed (including universities)
	Health care staff’s vacation’s cancelled
	All elective patient appointments, surgeries cancelled at Hacettepe University Hospital
17 March 2020	First death due to Corona in Turkey
20 March 2020	All meetings were cancelled
	Hacettepe Medical School started online education programme.
22 March 2020	Rotational working, working at home rules effective
	Stay-home decision for seniorn citizens (>65 years)
28 March 2020	All international travels are cancelled
04 April 2020	Travel ban to/from 31 cities
	Stay-home decision for younger generation (<20 years)
10 April 2020	Lock-down for weekend in 31 cities
14 April 2020	MoH announced any staff working in oncology departments should not be
	assigned to pandemic units
18 April 2020	Lock-down for weekend in 31 cities
23 April 2020	A four days lock-down during National Children’s day and weekend
01 May 2020	A three days lock-down during weekend and Labor day
11 May 2020	Re-opening of the shopping centres
23 May 2020	Four days national lock-down during Eid.
01 June 2020	All domestic travels are allowed again
	Restaurants, cafes, shops, museum, libraies, swimming pools reopened.
	All goverment officers returned to normal working hours.
	Nurseries re-opened
	Number of pandemia hospitals were started to decrease
	Normalisation of hospitals patient admission
	Concert hall re-opened at 50% capacity in accordance with pandemia plan
01 July 2020	Wedding halls, theatres, cinemas were re-opened
29 July 2020	MOH changed the reporting system of COVID-19 cases from daily number of the new “cases” (all test positive) to daily number of new ‘patients’ (only symptomatic cases)
01 September 2020	HES code (Life Fits Into Home) procedures were started. This is mobile phone app for COVID-19 contact tracing
12 October 2020	Face to face education in primary/secondary/ high schools were re-started nationally
11 November 2020	Smoking in crowded streets/squares were banned
21 November 2020	Lock-down for restricted hours at national level were re-started
	<20 years and >65 years were allowed to go out in limited time of the day
23 November 2020	Education in school was re-organised from ‘face to face’ to ‘online’ system
	All restaurants and cafes were closed for seating (takeaway and home delivery only)
01 December 2020	Nationwide lockdown was expanded as 21:00 pm to 05:00 am during weekdays and from 21:00 pm Friday to 05:00 am Monday for whole weekend

**Table 4.** Departmental actions for the COVID-19 prevention and continuation of the cancer care.

Real-time follow-up and compliance with the national and hospital regulations Regular follow-up of the global situation and learning from others Pandemia management team was established with the members of faculty members, chief nurse, paediatric haematooncology fellows and senior residents Routine daily meeting to monitor the situation Cancellation of face to face meeting and using the effective on-line tumour boards and training tools High level communication and informing of patients and care-givers High level communications among the health care staff Using surgical masks, physical/social distancing, PPE and hand washing Postponement of elective appointments including routine periodic check-up for off-therapy patients, imaging appointments, etc. Using the telephone call to respond the patients/caregivers according to their needs for emergent problems, treatment modification, appointment pospontment, planning for urgent appointment, etc. Doing a risk analyis for patients who are on treatment Not to cancel any appointment for patients on-therapy Diverting the patients who were not able to come to us, to nearest local facility Screening for all patients entering the department through a triage protocol brought by infection control committee COVID-19 PCR became a routine in paediatric oncology in-patient admission HES code (Life Fits Into Home) checking for all parents who are allowed to stay with their children at inpatient admissions was started. (This is mobile phone app for COVID-19 contact tracing at national level.) Sending the suspected and COVID-19 positive cases to the specialised area for further evaluation and care Continue with strict measurement on COVID-19 control
